# Integrating MAPK pathway inhibition into standard-of-care therapy for pediatric low-grade glioma

**DOI:** 10.3389/fonc.2025.1520316

**Published:** 2025-02-11

**Authors:** Erin E. Crotty, Aimee A. Sato, Mohamed S. Abdelbaki

**Affiliations:** ^1^ Ben Towne Center for Childhood Cancer and Blood Disorders Research and the Department of Pediatrics, Seattle Children’s Hospital, Fred Hutchinson Cancer Research Center, University of Washington, Seattle, WA, United States; ^2^ Division of Pediatric Neurology, Department of Neurology, Seattle Children’s Hospital, University of Washington, Seattle, WA, United States; ^3^ Division of Hematology, Oncology and Bone Marrow Transplant, St. Louis Children’s Hospital, Washington University School of Medicine, St. Louis, MO, United States

**Keywords:** pediatric low grade glioma, trametinib, selumetinib, MEK, MAPK pathways, BRAF, CNS tumors, LMIC

## Abstract

Pediatric low-grade gliomas (pLGG) are a group of tumors largely driven by alterations in a single genetic pathway, known as the RAS-RAF-mitogen-activated protein kinase (MAPK) pathway. Recent biologic insights and therapeutic targeting of MAPK-alterations have dramatically shifted the treatment approach in pLGG. While chemotherapy remains front-line therapy for unresectable pLGG in most scenarios (with the notable exception of *BRAF* ^V600E^-altered tumors), many patients recur following cytotoxic agents and require further treatment. Inhibitors of the MAPK pathway, primarily MEK and RAF kinase inhibitors, have emerged as effective and tolerable second-line or later therapy for pLGG. As familiarity with these targeted agents increases, their indications for use continue to expand and Phase 3 clinical trials investigating their utility in the front-line setting are ongoing. We have adopted mitigation strategies for their associated toxicities; skin toxicity, in particular, is now managed by prevention strategies and early dermatologic intervention. This review highlights current approaches for the clinical implementation of MEK and RAF kinase inhibitors for pLGG, focusing on the practical aspects of drug administration, toxicity management, response monitoring, and distribution to patients experiencing geographic or financial barriers to care. Additionally, we review important considerations for the off-label use of these agents while contemporaneous clinical trials assessing front-line efficacy are ongoing. We discuss the potential for more expansive or histology-agnostic tumor targeting using MEK inhibitors, harnessing their biologic relevance for other *RAS*-altered conditions.

## Introduction

Pediatric low-grade glioma (pLGG) represents the most common brain tumor in children, comprising up to 30-40% of all pediatric central nervous system tumors (1). The umbrella term, pLGG, encompasses a heterogenous group of tumors, with individual entities defined by their unique immunohistopathologic and molecular features. pLGG tumors are classified according to the 2021 World Health Organization (WHO) Central Nervous System (CNS) under 3 distinct categories: pediatric-type diffuse low-grade gliomas, circumscribed astrocytic gliomas, and glioneuronal or neuronal tumors (2). Among these, pilocytic astrocytoma (PA) is the most prevalent, making up 10-15% of all brain tumors in children and 5% of brain tumors in adults (1). The vast majority, nearly 70% of pLGG, harbor genomic driver mutations in the mitogen-activated protein kinase (MAPK) pathway, and therefore, pLGG is often considered a “single pathway disease.” (3) While pLGG can occur in any anatomic location in the CNS, distinct molecular drivers within the MAPK pathway often map to a particular CNS location. For instance, pleomorphic xanthoastrocytomas arise most commonly in the supratentorium and harbor *BRAF* ^V600E^ mutations, while PAs more frequently harbor *BRAF* fusions and arise in the posterior fossa or optic pathway. pLGGs are also commonly associated with neurofibromatosis type 1 (NF1), where optic pathway gliomas occur in 15-20% percent of patients (4, 5). Patients with NF1 carry a higher risk for the development of other RAS pathway-associated tumors (e.g. plexiform neurofibromas) and glial tumors, including brainstem gliomas and diffuse astrocytomas (6, 7).

## Current standard therapy

Surgical resection is the mainstay of front-line therapy for symptomatic pLGG; however, more than 50% of pLGGs occur in locations that are either not amenable to surgery or only amenable to a biopsy or limited resection (e.g. tumors located in the diencephalon, brainstem, optic pathway, or spinal cord) (8). Treatment for inoperable pLGG is largely determined by clinical symptoms, with gliomas in the optic pathway or brainstem often necessitating urgent initiation of therapy for vison preservation or neurologic symptoms.

Chemotherapy remains standard of care for inoperable or residual pLGG, with well-documented efficacy and tolerability in pediatric patients of all ages, even the very young. While the 5-year overall survival (OS) with chemotherapy ranges from 86-94% on recent studies, 5-year progression-free survival (PFS) is less favorable at 42-45% in pLGG, with slightly better PFS reported in patients with NF1 (5-yr PFS 69-85%) (9, 10). Chemotherapy regimens including carboplatin and vincristine or single-agent vinblastine are commonplace today, and while the adverse effect profile of these agents is relatively well tolerated, they carry risks of myelosuppression, gastrointestinal toxicity, and peripheral sensory and motor neuropathy (9–11). Carboplatin allergy or similar adverse reaction has been reported in up to 8-20% of patients with pLGG receiving combination carboplatin and vincristine (9, 10, 12, 13). Cranial irradiation is also very effective against pLGG and has demonstrated improved PFS compared to conventional chemotherapy (62% *versus* 42% 10-year PFS compared to carboplatin/vincristine on HIT-LGG-1996) (13); however, radiation therapy is largely avoided in most sporadic pLGG and almost all NF1-associated pLGG due to the risk of significant late effects, including secondary malignancy, neurocognitive impairment, hormonal dysfunction, growth delay, vasculopathy and cerebrovascular injury (14–17).

Regardless of the choice of upfront therapy, unresectable pLGG recurs in nearly 50% of patients after first-line therapy, and in many cases, requires episodic treatment throughout childhood and adolescence for periods of clinical progression (13, 18). The approach to multiply recurrent pLGG therapy is therefore reminiscent of a chronic condition, directed at periodic exacerbations and symptoms. Despite the relatively poor PFS of pLGG as a whole, the OS remains excellent (18–20). The current focus of our efforts to improve outcomes in pLGG is to identify agents that lead to a durable response and reduce morbidity. Targeted therapies, namely MAPK inhibitors that target RAF and MEK proteins, may fullfill that need. As the clinical use of MAPK inhibitors expands and duration of exposure increases, it will be critical to understand their side effects within the drug class and as individual agents, along with how to appropriately monitor tumor response. Herein, we review the practical aspects of MAPK inhibitor administration and toxicity management, as well as discuss implications for patients in limited resource settings. These oral agents carry inherent benefits for patients for whom access to a pediatric oncology facility is restricted, and while ongoing studies are designed to answer lingering questions regarding the safest, most effective front-line therapy for pLGG, the use of MAPK pathway inhibitors frontline in limited populations is also discussed.

## MAPK inhibitor therapy

### Molecular landscape

Alterations in the *BRAF* oncogene are some of the most described molecular variants found in cancer. Together, oncogenic *BRAF* aberrations result in constitutive activation of *BRAF* protein kinase activity, leading to downstream signaling that drives tumor growth and proliferation. All pLGG tumor subgroups have a documented driver mutation in the *BRAF* oncogene or within a cooperating protein along the MAPK pathway, namely the RAS, RAF, MEK, and ERK kinases. In the case of *BRAF* ^V600E^ hotspot mutations, the Val600Glu activating mutation functions as a monomer to promote ERK signaling. *BRAF* can also form rearrangements or pair with fusion partners where the N-terminal inhibitory domain of *BRAF* is replaced and the resulting dimer retains *BRAF* kinase signaling that drives aberrant expression independent of *RAS* signaling (21, 22). Among these genomic fusions, *KIAA1549::BRAF* fusions are the most common and are found in nearly 70-80% of pilocytic astrocytomas and 30-40% of all pLGG (18, 22–25). As such, novel agents targeting this ubiquitous fusion are highly sought after for therapeutic indications (**Table 1**).

**Table 1:** Targeted therapy for pediatric low-grade glioma harboring MAPK pathway alterations.

Drug Name	Target	Dose	Route	Formulation	Evidence in NF1	Evidence in pLGG
Dabrafenib	B-raf enzyme	Children <12 years: 2.625 mg/kg BID 12+ years: 2.25 mg/kg BID Max: 150 mg/dose	Oral	Capsule, Dispersible tablet	n/a	Phase II evidence: - 47% ORR when combined with dabrafenib in BRAF^v600E^-mutant pLGG (26)
Vemurafenib	B-raf enzyme	550 mg/m^2^ BID Max: 960 mg/dose	Oral	Tablet	n/a	Phase I evidence: - 32% ORR in BRAF^v600E^-mutant pLGG (27)
Binemetinib	MEK1/2	32 mg/m^2^ BID Max: 45 mg/30 mg in NF/dose	Oral	Tablet	Phase II evidence: - 43% PR (28)	Phase II evidence: - 50% PR with BRAF fusion - 69% in sporadic pLGG without BRAF (28)
Cobimetinib	MEK1/2	0.8 mg/kg tablet or 1.0 mg/kg suspension daily (3 wk on, 1 wk off) Max; 60 mg/dose	Oral	Tablet, Suspension	n/a	Phase I/II evidence: - 5% PR, 59% SD (29)
Mirdametinib	MEK1/2	2 mg/m^2^ BID (3 wk on, 1 wk off) Max: 4 mg/dsoe	Oral	Capsule, Dispersible tablet	Phase 2b evidence: -52% ORR in plexiform neurofibroma (30)	Phase I/II evidence: - 63% ORR (31)
Selumetinib	MEK1/2	25 mg/m^2^ BID Max: 50 mg/dose	Oral	Capsule	Phase I evidence: - 70% response rate in plexiform neurofibroma (32) Phase II evidence: - 40% sustained PR (19)	Phase I evidence: - 20% sustained PR (20) Phase II evidence: -36% sustained PR (19)
Trametinib	MEK1/2	< 6 years: 0.032 mg/kg daily 6+ years: 0.025 mg/kg daily Max: 2 mg/dose	Oral	Tablet, Suspension	Phase II evidence: - 61% ORR in plexiform neurofibroma (33)	Phase II evidence: - 47% ORR when combined with dabrafenib in BRAF v600E-mutant pLGG (26) - Retrospective evidence for monotherapy (34–36)
Tovorafenib	RAF kinase	380 mg/m^2^ PO weekly Max: 2 mg/dose	Oral	Tablet, Suspension	n/a	Phase II evidence: - 67% ORR by RANO-HGG and 51% ORR by RAPNO criteria (37)

Wk, weeks; NF, neurofibromatosis; ORR, Overall response rate; PR, partial response; SD, stable disease; n/a, not available.

A handful of agents targeting the MAPK pathway have demonstrated tolerability and efficacy in prospective studies of children and adolescents with relapsed or refractory pLGG (19, 20, 26, 30, 32, 34, 37–39). *BRAF* inhibitors (e.g. vemurafenib, dabrafenib) were among the first agents tested in pLGG in an attempt to replicate early successes seen in adult glioma and melanoma driven by *BRAF* ^V600E^ mutations, in addition to the particularly poor PFS in this pLGG subset (40, 41). While BRAF inhibitors showed remarkable efficacy in *BRAF* ^V600E^-altered pLGG (42), surprisingly, tumors harboring *KIAA1549::BRAF* fusions demonstrated paradoxical growth with these agents, a biologic phenomenon that has since been extensively modeled and molecularly described (21). *BRAF* inhibitors are now exclusively used in patients with *BRAF* ^V600E^ point mutations and are contraindicated in patients with *BRAF* fusions. Dabrafenib, was later tested in combination with trametinib, a MEK inhibitor, as first-line therapy for *BRAF* ^V600E^-altered LGG and demonstrated improved PFS compared to standard chemotherapy (PFS 20.1 *versus* 7.4 months; HR, 0.31) and a superior safety profile (26, 38). These early studies supported trials showing efficacy of this combination in non-CNS solid tumors and led to accelerated FDA approval in 2022 of the combination dabrafenib/trametinib for the treatment of progressive or metastatic solid tumors harboring *BRAF* ^V600E^ mutations. In 2023, the FDA approved the combination for the first-line treatment of pediatric patients 1 year of age and older with pLGG with a BRAF^V600E^ mutation (43).

MEK inhibitors, including selumetinib, trametinib, mirdametinib, binimetinib, and cobimetinib, provide active inhibition of ERK signaling by targeting MEK, a kinase downstream from *BRAF* in the MAPK pathway. Several have been studied in pLGG, with demonstrated safety and efficacy data available in the recurrent pLGG and NF1-associated pLGG populations, and selumetinib is currently in clinical trials for newly diagnosed patients (NCT03871257, NCT04166409). The following discussion focuses on agents with published phase 2 trial data in pLGG and favorable toxicity profiles, with selumetinib and trametinib available for prescription off-label, leading to frequent real-world use as second-line therapy.

Selumetinib, a selective, oral small molecule inhibitor of MEK1/2, has shown a 40% sustained response rate (complete or partial response) in a phase 2 trial of children with progressive PAs harboring *KIAA1549::BRAF* fusions or *BRAF* ^V600E^ mutations (19). PFS at 2 years was 70% in *BRAF*-altered non-NF1 associated pLGG and 96% in patients with germline NF1. Patients with NF1-associated plexiform neurofibromas have similarly shown response rates around 70% to treatment with single-agent selumetinib and it is now FDA-approved for that indication (32). Selumetinib is currently being compared to upfront standard chemotherapy in two phase 3 clinical trials for patients with newly-diagnosed pLGG, both with and without NF1 (NCT03871257, NCT04166409).

Trametinib is another potent, oral small molecule inhibitor of MEK1/2 with non-competitive ATP binding properties. While large, prospective trials are still ongoing (NCT03363217), retrospective analyses show promising objective response rates in recurrent pLGG, supporting drug class efficacy in this population (34–36). Interim results of a phase 2 study of single-agent trametinib demonstrated an overall response rate of around 47% (complete, partial, or minor response) in pLGG and a 60% response rate using volumetric assessments in patients with NF1-associated plexiform neurofibroma (33, 44). Trametinib was FDA approved in 2013 for adult *BRAF* ^V600E^-altered melanoma and has been used commonly off-label in pediatric patients as molecularly-guided therapy for recurrent or refractory pLGG.

Mirdametinib is another highly brain penetrant inhibitor of MEK1/2 that is currently being investigated as monotherapy in a phase I/II trial to assess safety and preliminary efficacy for relapsed or refractory pLGG (NCT04923126). To date, 12/19 (63%) patients achieved promising objective responses (1 major, 6 partial, 5 minor). The phase 2 component is ongoing to establish safety and efficacy, including in newly diagnosed patients and those with prior exposure to MEK inhibitors (31, 45).

Tovorafenib, in contrast to the MEK inhibitors discussed above, is an oral, highly selective, type II RAF inhibitor that targets both mutant and wild-type A-Raf, B-Raf, and C-Raf protein kinases. Type II RAF inhibitors effectively inhibit RAF dimers, including BRAF fusions, in addition to BRAF monomers for pan-RAF inhibitory function. Among 137 patients enrolled on a phase 1 trial in *BRAF*-altered relapsed or refractory pLGG, tovorafenib showed an overall response rate (including minor responses) of 51% by RAPNO criteria and 67% by RANO-HGG criteria (primary endpoint), including 12 (17%) patients with a complete response and a median duration of response of 13.8 months (37). Patients entered the trial having received a median of three lines of prior therapy, with 61% previously treated with a MEK and/or BRAF inhibitor. The FDA granted tovorafenib accelerated approval for recurrent or refractory pLGG in 2024 (46). Tovorafenib is currently being compared to standard-of-care chemotherapy regimens, including single-agent vinblastine or carboplatin/vincristine in newly diagnosed RAF-altered pLGG (37).

### Toxicity management

**Figure 1:**
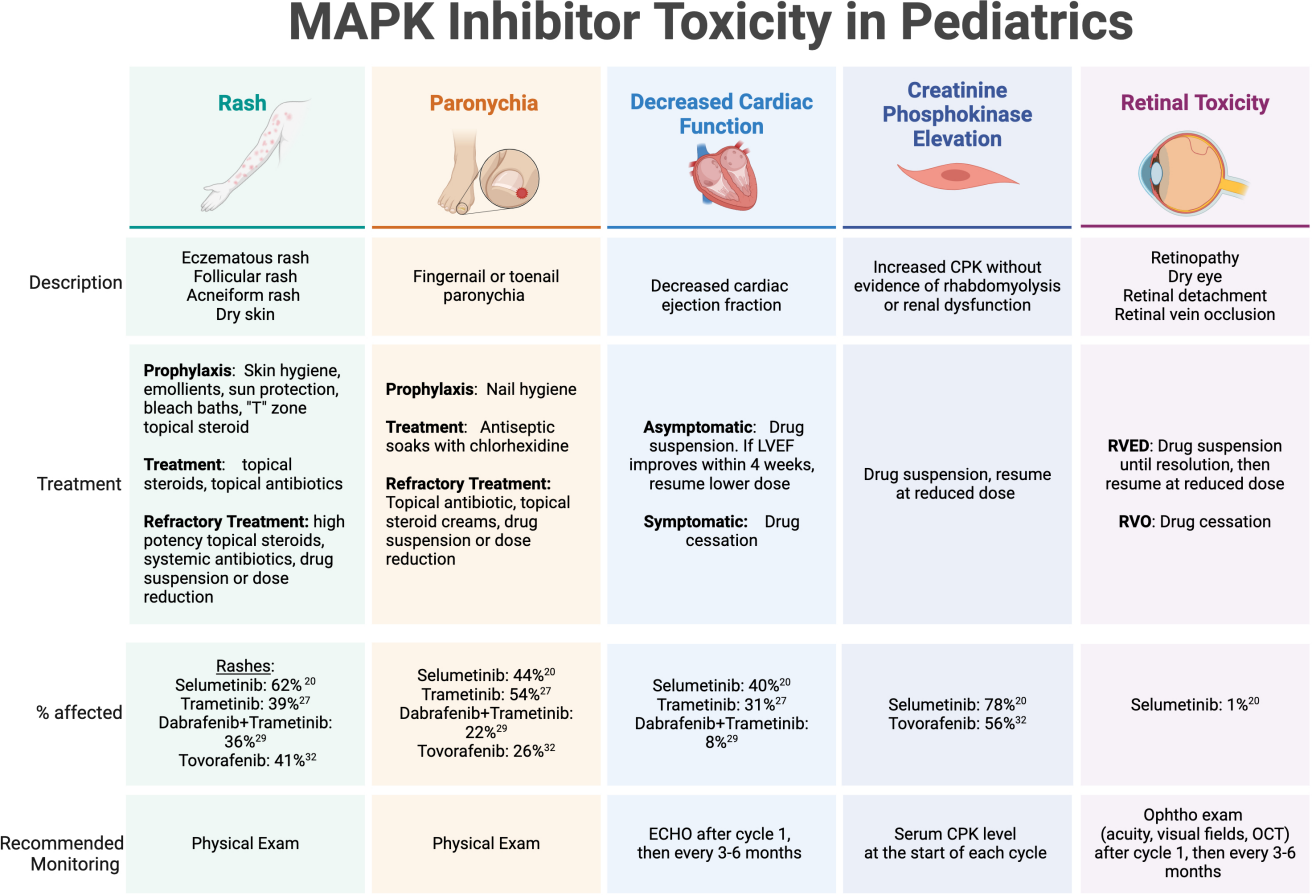
MAPK inhibitor toxicity in pediatrics. Select toxicities with high incidence or potential severity reported on published trials of MAPK inhibitors in children and adolescents with recurrent or refractory pLGG. LVEF, left ventricular ejection fraction; CPK, creatinine phosphokinase; RVED, Retinal Vascular Endothelial Dysfunction; RVO, retinal vein occlusion; Ophtho, ophthalmology.

MAPK inhibitor toxicities have been well described in pediatric populations (**Figure 1**). The most common treatment-related adverse events include dermatologic manifestations (rash, paronychia, photosensitivity, hair color change), gastrointestinal toxicity (nausea, vomiting, diarrhea), ophthalmologic toxicity (serous retinopathy, retinal vein occlusion, retinal detachment), cardiac complications (decreased ejection fraction), asymptomatic creatinine kinase elevation, weight gain, and fatigue (47). Recently, cooperative groups have identified a need to develop clinical guidance for practitioners for managing frequently encountered side effects, especially regarding dermatologic recommendations due to the frequency and occasional severity of MAPK inhibitor-associated rashes (48). One such advisory board from the Children’s Tumor Foundation has proposed and implemented recommendations for toxicity management and side effect monitoring for patients with NF1 receiving MEK inhibitors, which has now been widely adopted (49).

The dermatologic manifestations of MAPK-targeted therapy can range from eczematous and acneiform rashes to paronychia and wound complications. Recommended strategies for prevention and management include symptom-related measures applied to specific dermatologic conditions. For rashes, consensus guidelines include prophylactic measures, such as applying skin moisturizers/emollients to maintain an optimal skin barrier, sun protection, and bleach baths to minimize skin flora and reduce the risk of infectious complications and pruritis. In adolescent patients, topical antibiotics and low dose topical steroids are recommended for prevention of facial acne in the “T” zone. Once a rash is present, treatments include topical steroids, topical and oral antibiotics, and consideration of drug suspension until resolution or significant improvement. Paronychia is also pervasive in patients on MEK and RAF inhibitors, and mild cases can be addressed with frequent antiseptic soaks using chlorhexidine, topical antibiotics, and topical steroids. Dermatology specialty care is recommended for persistent or severe cases of skin toxicity. Wound healing complications have been less frequently reported, but are a potential cause of morbidity in patients who undergo surgery while on therapy, which may include procedures for the placement of a ventricular shunt catheter for CSF diversion or a central venous access line. Primary incisions or skin ulcerations caused by pressure wounds may have longer healing times and require wound care expertise for appropriate management.

While the rates of cardiac toxicity are low in children receiving MAPK pathway inhibitors in published reports, most guidelines recommend routine echocardiograms (ECHO) to monitor for asymptomatic diminishing ejection fraction (EF) while on MEK inhibitor therapy. Selumetinib, for example, has been associated with a decrease in EF >10% below the normal range in a single pediatric patient with NF1-associated pLGG and 20 (40%) patients with non-NF1 associated pLGG (19, 32). Most of the limited reported cases of EF dysfunction were detected on routine screening, but even for asymptomatic decreases beyond 10% of baseline function, the consensus recommendation is to hold MEK inhibitor therapy and discontinue altogether if no improvement within 4 weeks off therapy since EF decrease is generally reversible after drug suspension or discontinuation. Routine ECHO monitoring is recommended after 1 month of therapy, then at routine intervals (e.g. 3-6 months) thereafter. It is worth noting that tovorafenib has not been associated with cardiac adverse effects.

Ocular adverse events are fortunately rarely reported in pediatric patients on published studies of MAPK inhibitors. However, when present in adult patients, they can result in significant visual complications and even blindness (50, 51). Considered a class effect that affects anywhere from 5-90% of adult patients treated with MEK/BRAF inhibitors based on published reports (51), retinopathy can present with symptomatic blurry vision, floaters, or photophobia, and most severely, may result in retinal detachment or retinal vein occlusion. MEK inhibitor-associated retinopathy (MEKAR) is generally self-limited and may resolve with or without drug cessation (50). Recommendations for monitoring vary considering that visual symptoms are normally present and therefore can be monitored clinically, however most practitioners recommend an ophthalmologic exam early into therapy, usually after 1 month, then at routine intervals (e.g. 3-6 months) thereafter (49). The ophthalmology exam should include visual acuity at a minimum, visual field testing in patients with optic pathway gliomas, and consideration of optical coherence testing (OCT) for detection of small retinal detachments by direct examination of the retina and choroid. Tovorafenib has not been associated with retinal or ocular-related adverse effects.

Interestingly, tovorafenib can lead to growth arrest – a side effect that hasn’t been reported with any of the MEK inhibitors, and it is unknown whether this particular toxicity is considered a class effect. Preliminary data on tovorafenib shows pediatric and adolescent patients demonstrate growth arrest on therapy without advancement of bone age or immature closure of growth plates (37, 52). This adverse effect reverses once drug therapy is stopped and patients have resumed expected annualized growth velocities after therapy, with some exceeding the expected average post-treatment (52).

Data on long-term toxicities or late effects of MAPK inhibitors are immature, owing to the limited amount of time they have been commercially available, which is a disadvantage to these drugs compared to chemotherapy, however, off therapy toxicity data is actively being collected. Organ function, particularly cardiac and ocular function, along with physiological effects on growth, puberty, and visual and neurocognitive outcomes are being monitored.

### Response monitoring

The objective criteria used for monitoring treatment response to MAPK inhibitor therapy has been through several iterations. On the phase I trial of selumetinib in patients with recurrent pLGG, objective response was initially measured by including the enhancing tumor components on T1 post-contrast magnetic resonance imaging (MRI). This was later amended to incorporate more widely accepted definitions of response in pLGG, which does not rely on changes in the enhancement pattern over time, as these are known to increase/decrease without intervention, deviate from tumor volume measurements, and are not prognostic (20). Instead, T2/fluid attenuated inversion recovery (FLAIR) sequences were used to more accurately define tumor response using bidimensional measurements and these were carried forward for objective response assessments on the phase 2 selumetinib trial (19).

Response assessment on the phase 2 trial of tovorafenib in recurrent pLGG utilized the Response Assessment in Neuro-Oncology High-Grade Glioma (RANO-HGG) criteria as a primary endpoint, as these were considered the only validated criteria by the FDA at the time of trial initiation (37). To address the same concerns about inclusion of enhancement pattern as a defining feature, secondary assessment of response was performed by Response Assessment in Pediatric Neuro-Oncology Low-Grade Glioma (RAPNO) criteria and Response Assessment in Neuro-Oncology Low-Grade Glioma (RANO-LGG), both of which more accurately characterize disease burden in pLGG. The ongoing phase 3 trial of tovorafenib *versus* standard chemotherapy utilizes RANO-LGG criteria, with alternative imaging criteria as secondary objectives and endpoints.

In addition to objective tumor response, it is important to understand MAPK therapy-related effects on chronic morbidities associated with pLGG, including functional outcomes like vision and neurocognitive development. While traditional chemotherapy may stabilize vision, published data on MEK inhibitors suggest that these agents may have further benefit by improving visual acuity (19, 26, 37). To determine an accurate risk-benefit ratio of newer agents, ongoing studies are thoughtfully addressing these measures and including quality of life (QOL) and patient-reported outcomes (PRO) assessments, adaptive behavior tools, daily living domain scores, communication assessments, additional visual assessment tools, and neuropathy scores. In addition, the clinical benefit rate, time to response, and duration of response are adding qualifying data to our standard measures of tumor response.

MAPK targeted therapies have an established role in the treatment of relapsed or refractory pLGG following chemotherapy as second-line or later therapy, and may better accommodate episodic use in a way that cytotoxic agents cannot. For example, retreatment with the MEK inhibitor selumetinib has been shown to be effective for tumor control in melanoma and may suggest drug resistance to these agents is reversible, unlike other small molecule inhibitors used in cancer (53). In one study. discontinuing treatment with BRAF inhibitors (dabrafenib or vemurafenib) in BRAF^V600E^ -mutated pLGGs resulted in rapid regrowth in 76.5% of tumors, however 90% of pLGGs responded if rechallenged with BRAF inhibition alone or when combined with MEK inhibition (54). This retreatment phenomenon draws a stark contrast to chemotherapy regimens, which are generally not re-used at progression, and has implications for how duration of response should be measured on targeted therapies.

## Administration and access for special populations

Compared to standard first-line chemotherapy, oral agents may have inherent benefits for minimizing the burden of disease in patients with pLGG. The MAPK-targeted therapies described above are administered orally, with most (except selumetinib) available as a liquid formulation or dispersible tablet ideal for young children and patients requiring enteral nutrition. Based on pharmacokinetic studies, MEK and BRAF inhibitors are given daily (once to twice per day), with some MEK inhibitors requiring an empty stomach for optimal absorbtion (45, 55). Tovorafenib can be taken with or without food and is administered once weekly. Beyond convenience, oral MAPK inhibitors may also reduce financial toxicity for patients living in low resource or geographically limited areas, since on therapy monitoring necessitates fewer ambulatory office visits compared to a chemotherapy regimen administered weekly. Additionally, unlike chemotherapy, MAPK inhibitors are not associated with serious infectious complications since they do not cause significant myelosuppression or require central venous access, so unexpected hospital admissions are rare. While few studies have fully characterized the long and short-term treatment-related morbidities associated with pLGG treated with standard chemotherapy and surgery, a recent retrospective study found a surprisingly higher level of healthcare resource utilization (HRU) and symptom burden in this population than has previously been captured (16, 56). These data and the data currently being captured in ongoing clinical trials assessing QOL and PRO will be invaluable in establishing treatment guidelines and assessing whether a front-line oral regimen may substantially improve cost burden, psychosocial impact, and access to care in pLGG.

Ambulatory infusion centers capable of delivering intravenous chemotherapy to pediatric patients are limited, and pediatric neuro-oncology programs with clinical expertise in pLGG even more so. In a recent study evaluating geographic access to pediatric cancer care, the median travel times to a pediatric oncology center were longest for American Indian (AI) or Alaska Native (AN) pediatric populations (46 [range16-104] minutes) and residents of rural areas (95 [range 68-135] minutes) (57). These data provide additional evidence that AI and AN communities continue to endure health disparities and that geographic-based social determinants of health may be a significant contributing factor. A real-world example of this is experienced by patients living in rural Alaska, wherein weather patterns may routinely delay or prevent travel in and out of community villages and a single pediatric oncology center exists to serve the entire state, encompassing a large geographic region. Historically, patients with pLGG requiring therapy beyond surgery have had to relocate, often moving hundreds of miles away for the duration of treatment. As discussed above, chronic episodic therapy for multiply recurrent disease only adds to the immense financial and psychosocial burden of treatment on the family and larger community. Alaska is just one example of many locations in the United States with high Area Deprivation Index (ADI) scores that may benefit from rethinking our current front-line strategy for pLGG in context of a larger goal toward health equity in pediatric cancer care.

**Figure 2:**
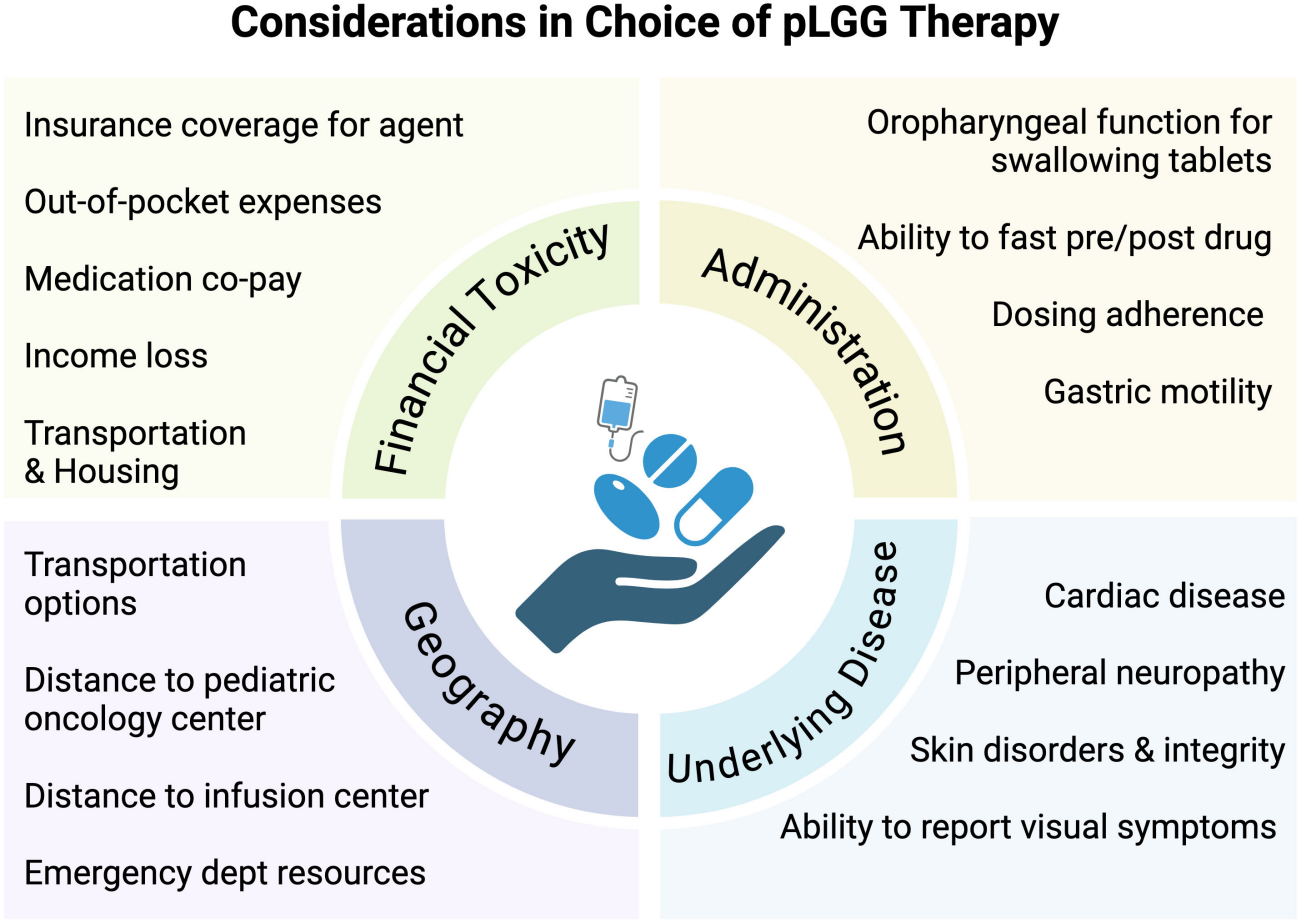
Considerations in choice of pLGG therapy. Schema depicting psychosocial, economic, geographic, financial, and disease-related considerations in determining treatment decisions in pediatric low-grade glioma.

With the emergence of telehealth capabilities, providers are better equipped with infrastructure to deliver specialized cancer care remotely and appropriately monitor therapy-related toxicities. Oral regimens may require fewer in-person evaluations and infrequent tests performed by ancillary services (e.g. cardiology, ophthalmology), allowing much broader access to care by reducing travel burden and financial toxicity. This would allow patients to receive therapy while living further away from pediatric brain tumor programs. The difference in financial toxicity between weekly and monthly, or even every 3-month travel to and from a healthcare facility alone is noteworthy. In addition, drugs can now be delivered by mail-in specialty pharmacies, reducing the strain on local healthcare systems. While expanding clinical sites may not be feasible, expansion of existing infrastructure and a thoughtful approach to our clinical care standards for at risk populations may go a long way in improving health outcomes (**Figure 2**).

In low- or middle-income countries (LMIC), access to MAPK inhibitors is even more restricted, and is impacted by drug availability and pricing. Targeted therapies are not available in many LMIC countries, and when available, there are significant limitations related to regional drug supply or their very high cost (58, 59). In addition, molecular testing for identifying MAPK alterations is limited in these settings, impacting the identification of patients who are likely to benefit from targeted therapies. In an effort to improve outcomes for children with cancer around the world, the World Health Organization (WHO) launched a Global Initiative for Childhood Cancer (GICC) under its Cure*All* Framework and included pLGG as one of six “tracer cancers” to monitor success of the program (60). Medicine pricing and procurement is among the pillars of the program, and requires both government and industry partnerships, however it will be equally critical to evaluate its success in co-establishing a infrastructure for molecular diagnosis in LMIC settings.

### Considerations for balancing research integrity with off-label use

Off-label use of MAPK pathway inhibitors, namely their use as first-line therapy in newly diagnosed patients with pLGG, is controversial. “Off-label” typically entails the use of cancer therapeutics in unapproved populations and is a common practice in pediatric oncology generally, since pediatric and adolescent patients are almost always excluded from registrational FDA trials and there is often sound scientific evidence and safety data to support their use (61). Practitioner comfort with MAPK inhibitor prescribing has increased with the accelerated FDA approvals of selumetinib, dabrafenib/trametinib, and tovorafenib, along with the publication of phase I/II trial safety and PK data. However, this has coincided with the opening of phase 3 clinical trials assessing front-line efficacy of selumetinib *versus* carboplatin/vincristine in pediatric patients with NF1 (NCT03871257) and without NF1 (NCT04166409), and tovorafenib *versus* standard chemotherapy in non-NF1 associated pLGG (NCT05566795). Prior to the results of these trials and maturation of the longer-term data on late effects, the use of MAPK inhibitors as front-line therapy raises important ethical considerations. The research community has prioritized answering critical questions about efficacy (non-inferiority compared to standard of care chemotherapy), long-term safety, and functional outcomes that would not be captured outside the context of a clinical trial. Each of these facets is essential to fully characterize the clinical benefit of MAPK targeted therapy, as compared to our current standard, and will be invaluable in developing much needed treatment guidelines.

While the vast majority of patients with pLGG can undergo study randomization to a chemotherapy regimen or MAPK inhibitor without undue burden, the special populations described above, including patients with geographic or financial stress, may face additional barriers. For these patients, many factors may preclude study enrollment and in order to facilitate patient-centered care, many providers offer front-line, off-label use of MEK or RAF inhibition. The literature on this type of prescribing is lacking and would benefit from dedicated efforts to further characterize which specific patient populations may be candidates for alternative upfront therapy for pLGG.

## Future directions

Given the ubiquitous nature of MAPK pathway alterations in disease and chronic syndromes beyond cancer, researchers are now exploring new target populations that may benefit from MAPK targeting. MEK inhibitors are being investigated, and in some cases prescribed as first-line therapy, in NF1 and other RASopathy syndromes, including Noonan syndrome, hypertrophic cardiomyopathy, multifocal atrial tachycardia (MAT), lymphatic anomalies, capillary–arteriovenous malformations, Kaposiform lymphangiomatosis, and cardiofaciocutaneous syndrome, among others. While MEK inhibitors are effective in treating pLGGs in those with NF1, consensus remains that they are best used within a clinical trial or for relapsed disease (62). However, with several applications of MEK inhibitors for complications in NF1, there may be more interest in utilizing MEK inhibitor as first line-therapy in circumstances when multiple disease types may be addressed with single agent therapy, such as a MEK inhibitor for simultaneous pLGG and plexiform neurofibroma treatment.

The lessons learned from our early BRAF inhibitor experience serve as a reminder that not all RAS alterations are alike and target inhibition can have paradoxical effects in certain biologic conditions. As the use of MAPK inhibition expands for rare subsets of patients, it will be critical to report early experiences, including unexpected toxicities. Additionally, in our current molecular area as we discover more genomic alterations that span disparate tumor types, such as BRAF^v600E^, which can be seen in melanoma, glioma, and colon cancer, there may be an expanded role for tumor-agnostic drug approvals for targeted therapies.

## Conclusion

Treatment guidelines are lacking on how best to incorporate targeted therapy into standard practice for pLGG. As familiarity and availability of MEK and RAF inhibitors increases, we have an opportunity to provide early, evidence-based support for practitioners on how and when to use these agents towards the goal of improving outcomes and reducing morbidity. While essential phase 3 clinical trials are ongoing, all patients should be offered opportunities to participate in research and the use of MAPK inhibition as first-line therapy (beyond BRAF^v600E^-altered tumors) reserved for judicious use in special populations where financial or geographic barriers exist.
